# Cardiovascular Health Benefits of Specific Vegetable Types: A Narrative Review

**DOI:** 10.3390/nu10050595

**Published:** 2018-05-11

**Authors:** Lauren C. Blekkenhorst, Marc Sim, Catherine P. Bondonno, Nicola P. Bondonno, Natalie C. Ward, Richard L. Prince, Amanda Devine, Joshua R. Lewis, Jonathan M. Hodgson

**Affiliations:** 1 School of Medical and Health Sciences, Edith Cowan University, Joondalup, WA 6027, Australia; marc.sim@ecu.edu.au (M.S.); c.bondonno@ecu.edu.au (C.P.B.); a.devine@ecu.edu.au (A.D.); joshua.lewis@ecu.edu.au (J.R.L.); jonathan.hodgson@ecu.edu.au (J.M.H.); 2 Medical School, Royal Perth Hospital Unit, The University of Western Australia, Perth, WA 6000, Australia; natalie.ward@curtin.edu.au; 3 School of Biomedical Sciences, Royal Perth Hospital Unit, The University of Western Australia, Perth, WA 6000, Australia; nicola.bondonno@uwa.edu.au; 4 School of Public Health & Curtin Health Innovation Research Institute, Curtin University, Bentley, WA 6102, Australia; 5 Medical School, Queen Elizabeth Medical Centre Unit, The University of Western Australia, Nedlands, WA 6009, Australia; richard.prince@uwa.edu.au; 6 Department of Endocrinology and Diabetes, Sir Charles Gairdner Hospital, Nedlands, WA 6009, Australia; 7 Centre for Kidney Research, Children’s Hospital at Westmead, Westmead, NSW 2145, Australia; 8 School of Public Health, Sydney Medical School, University of Sydney, Sydney, NSW 2006, Australia

**Keywords:** vegetables, leafy green, cruciferous, allium, yellow-orange-red, legumes, carotenoids, organosulfur compounds, nitrate, cardiovascular diseases

## Abstract

Adequate vegetable consumption is one of the cornerstones of a healthy diet. The recommendation to increase vegetable intake is part of most dietary guidelines. Despite widespread and long-running public health messages to increase vegetable intake, similar to other countries worldwide, less than 1 in 10 adult Australians manage to meet target advice. Dietary guidelines are predominantly based on studies linking diets high in vegetables with lower risk of chronic diseases. Identifying vegetables with the strongest health benefits and incorporating these into dietary recommendations may enhance public health initiatives around vegetable intake. These enhanced public health initiatives would be targeted at reducing the risk of chronic diseases, such as cardiovascular diseases (CVD). Specific vegetable types contain high levels of particular nutrients and phytochemicals linked with cardiovascular health benefits. However, it is not clear if increasing intake of these specific vegetable types will result in larger benefits on risk of chronic diseases. This review presents an overview of the evidence for the relationships of specific types of vegetables, including leafy green, cruciferous, allium, yellow-orange-red and legumes, with subclinical and clinical CVD outcomes in observational epidemiological studies.

## 1. Introduction

Poor diet is a major contributor to the risk of chronic diseases. Chronic diseases, including cardiovascular diseases (CVD), cancer, chronic respiratory diseases and diabetes, account for 90% of all deaths in Australia [1] and 70% of all deaths worldwide [2]. Of these, CVD remains the number one cause of death [2,3,4]. There is abundant evidence supporting public health practice promoting increases in vegetable intake [5,6,7], which is the cornerstone of dietary approaches for chronic disease primary prevention. Despite widespread and long-running health promotion messages to increase vegetable intake [8], the majority of the world’s population have low vegetable intakes [9,10,11].

Current dietary guidelines promote an increase in intake of all vegetables [5,6,7]. These guidelines are mainly based on epidemiological evidence linking diets high in vegetables with lower incidence of chronic disease [12], and are supported by data suggesting that individual nutrients found abundantly in vegetables are protective [13,14,15]. Some vegetables may have substantially larger health benefits in comparison to others, implying not all vegetables are the same [16,17]. Therefore, the improvement of dietary guidelines to include targeted advice on consuming specific types of vegetables may enhance population health approaches to increase vegetable intake.

This narrative review focuses on the cardiovascular health benefits of specific vegetable types in observational epidemiological studies. Vegetables can be classified according to their chemical constituents or by biological classifications based on similarities such as evolutionary relationships. Frequently, these classification systems categorize similar vegetables together. Types of vegetables discussed in this review include leafy green, cruciferous, allium, yellow-orange-red and legumes.

## 2. Nutrients and Phytochemicals in Vegetables

**Figure 1:**
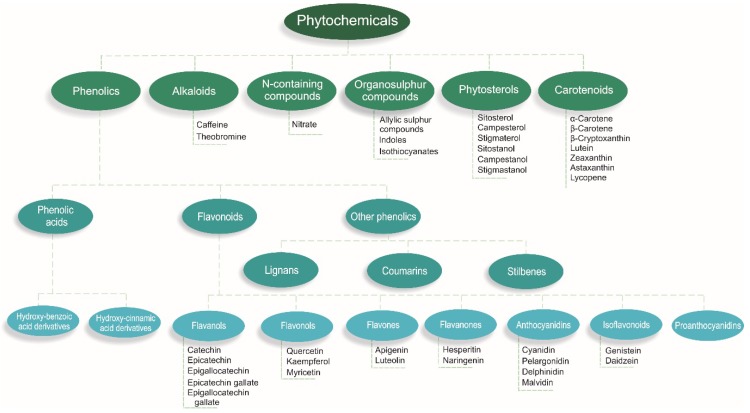
Classification of phytochemicals [18,22].

Vegetables contain many nutrients and phytochemicals that have been proposed to have cardiovascular health benefits [18]. Phytochemicals are compounds found in plant-based foods that have previously been considered non-essential components of the diet [19]. However, emerging evidence suggests phytochemicals extend benefits beyond that of basic nutrients and may be a vital part of a healthy diet [18]. Phytochemicals are produced by plants to reduce the risks of environmental stresses, such as insect attacks; however, when consumed by humans, they may exhibit a wide range of health benefits [19]. While over 20,000 phytochemicals have been identified in plant foods [20], a large percentage remain unknown or are poorly understood [21]. Many of these phytochemicals have been isolated to build an understanding of the benefits towards human health [18,22]. For the classification of phytochemicals, see Figure 1.

Meta-analyses have shown nutrients, such as dietary fiber [13], magnesium [15] and potassium [14], and phytochemicals, such as flavonoids [23] and carotenoids [24,25,26,27], are associated with benefits on cardiovascular health. Evidence also suggests cardiovascular health benefits of other nutrients, such as vitamin K [28] and vitamin C [29], and phytochemicals, such as nitrate [30,31] and organosulfur compounds [32]. These nutrients and phytochemicals may protect against CVD by a number of mechanisms. These include modulating enzyme activity; altering gene expression and signaling pathways; regulation of blood pressure; regulation of lipid and glucose metabolism; influence on antioxidant, anti-inflammatory, and antiplatelet activity; effects on endothelial function; and attenuation of myocardial damage [33]. In particular, carotenoids which are phytochemicals found abundantly in yellow-orange-red and dark green leafy vegetables [34], have been postulated to reduce oxidative stress and inflammation. This may be achieved by influencing transcription factors, such as nuclear factor κB (NFκB) or nuclear factor erythroid 2-related factor 2 (Nrf2), and their downstream targets, such as interleukin 8 (IL-8) and prostaglandin E2 (PGE2) or heme oxygenase (Hmox-1) and superoxide dismutase (SOD), respectively [35].

Strong evidence suggests nitrate, found abundantly in leafy green vegetables, beetroot and radish [36], can increase nitric oxide (NO) via the enterosalivary nitrate-nitrite-NO pathway. Nitric oxide is a cell signaling molecule important for vascular homeostasis [37]. Nitric oxide is involved in many physiological and pathological processes such as relaxing vascular smooth muscle tissue, increasing regional blood flow, and inhibiting platelet and leukocyte adhesion to vessel walls [38]. A combination of all these physiological benefits are likely to slow the progression of atherosclerosis. Evidence suggests organosulfur compounds may also slow the progression of atherosclerosis through anti-inflammatory and antiplatelet effects [39,40].

Organosulfur compounds are organic sulfur-containing compounds often associated with their foul odours. Glucosinolates, a class of organosulfur compounds, are found almost exclusively in cruciferous vegetables such as cabbage, broccoli, kale and Brussels sprouts. Anti-inflammatory properties of isothiocyanates, a breakdown product of glucosinolates, have been proposed through activation of the redox-sensitive transcription factor Nrf2, which control the expression of antioxidant and phase II enzymes [39]. Furthermore, studies have demonstrated in vitro inhibition of platelet aggregation of thiosulfinates, a class of organosulfur compounds found in allium vegetables such as garlic, onions, shallots, leeks and chives [40].

## 3. Classification of Vegetables

Large observational epidemiological studies have classified vegetable types in different ways. This is usually related to countries having varied accessibility and availability of different vegetable types [41]. Some large epidemiological studies have investigated individual vegetables, whilst others have investigated vegetable types grouped together based on botanical families, colors, or plant parts (e.g., stems and stalks, and leaves) [12,41]. The botanical classification of vegetables is based on physiological characteristics and include vegetables grouped under their botanic family name. Examples include Amaryllidaceae (garlic, leek, onion, and scallion), Chenopodiacea (beet and beet greens, spinach, and Swiss chard), Cruciferae/Brassica (broccoli, Brussels sprouts, cauliflower, Chinese broccoli, Chinese cabbage, collards, and kale) and Leguminosae (green peas, kidney beans, lentils, green snap beans, snowpeas, and soybeans) [41]. Color classification reflects the pigments of vegetable plant tissues and can also reflect the presence of phytochemicals such as beta-carotene (deep orange), anthocyanidin (red) and chlorophyll (green) [41]. Plant part classification is based on the edible part of the vegetable, including stems and stalks, leaves, legumes, bulbs, and roots and tubers. Stem and stalk vegetables, such as celery, are predominately high in dietary fiber due to their supported structure [41]. Leaves of vegetables are the most metabolically active and tend to be the most nutritious part of the vegetable being a good source of folate, carotenoids, vitamin C, flavonols, iron, zinc, calcium and magnesium [41]. Legumes are a good source of protein, starch, isoflavones, vitamin B6, folate and iron [41]. Bulbs are a good source of organosulfur compounds, in particular allicin [41]. Roots and tubers, such as potatoes, are a good source of resistant starch which may help maintain a healthy gut [42]. Gut dysbiosis has been shown to be associated with intestinal inflammation and has been linked to the development of CVD [43].

It has been suggested that grouping vegetables based on food components of public health significance is a way for nutrition professionals to explore health benefits [41]. Groupings often include dark green leafy vegetables; cabbage family vegetables; lettuces; allium family bulbs; legumes; deep orange/yellow roots and tubers; and tomatoes and other red vegetables. Despite the comprehensiveness in the development of this grouping system, there are some limitations. One limitation is that vegetable components, such as nitrate and organosulfur compounds, are not taken into consideration. In addition, observational cohort studies are restricted to specific groupings due to limited vegetable items on food frequency questionnaires.

**Figure 2:**
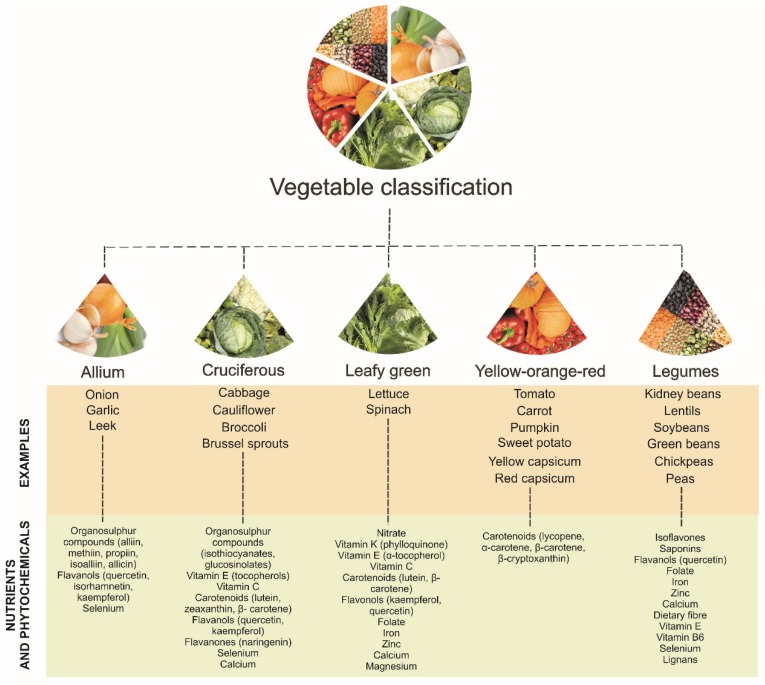
Classification of vegetable types with nutrients and phytochemicals associated with each vegetable type [24,25,26,32,41,44,45,46,47,48,49,50,51,52,53,54,55].

The following sections discuss the associations of specific vegetables types, including leafy green, cruciferous, allium, yellow-orange-red and legumes, with subclinical and clinical CVD outcomes. The aforementioned vegetable types are based on the above groupings of vegetables as well as dietary guidelines around the world, and have been modified slightly to align with specific phytochemicals found abundantly in these vegetable types. These include nitrate (leafy green vegetables) [44,45]; organosulfur compounds such as glucosinolates (cruciferous vegetables) [46,47] and cysteine sulfoxides (allium vegetables) [32]; carotenoids such as lycopene and bete-carotene (yellow-orange-red vegetables) [24,25,26,48]; and polyphenolic compounds such as isoflavones and saponins (legumes) [49]. For more information on other nutrients and phytochemicals associated with these vegetable types, see Figure 2.

## 4. Subclinical Measures of Atherosclerosis

Atherosclerosis, the underlying cause of CVD, is a complex multifactorial disorder of the arteries initiated by endothelial dysfunction, inflammation and dyslipidemia [56,57]. Carotid artery intima-media thickness (IMT) and focal plaques are subclinical measures of atherosclerosis, both of which have been shown to predict CVD outcomes [58,59,60]. Although carotid artery IMT and focal plaques are both interrelated, they may reflect different biological aspects of atherogenesis [61]. Carotid artery IMT captures both the atherosclerotic process as well as the compensatory thickening of the carotid wall in response to ageing and hypertension [62], and is strongly related to ischemic stroke [61]. Carotid focal plaques capture the atherosclerotic process, are dependent on the influx of lipids into the plaque, and are more related to hyperlipidemia and myocardial infarction [61].

Diets rich in vegetables, such as the vegetarian diet and the Mediterranean diet, have been shown to be associated with lower carotid artery IMT [63,64] and delayed progression of atherosclerotic plaques [65]. Very few observational cohort studies have investigated the relationships of vegetable intake alone and/or specific vegetable types with subclinical measures of atherosclerosis [66]. To our knowledge, we are the first to publish an association of total vegetable intake, and in particular, intake of cruciferous vegetables, with carotid artery IMT [67]. However, no relationship between total vegetable intake and carotid atherosclerotic plaque was observed. This could be due to carotid artery IMT and carotid focal plaques differing in biological aspects of atherogenesis as described above.

## 5. Cardiovascular Disease Clinical Endpoints

Increasing vegetable intake is widely recommended for reducing CVD risk, along with other chronic disease. These recommendations are incorporated into dietary guidelines around the world [5,6,7] and are primarily based on large-scale prospective cohort studies linking diets high in vegetables with lower chronic disease risk. The amount of vegetable intake recommended in dietary guidelines vary globally, but is usually around 5–6 servings/day (375–450 g/day). More than 60 prospective cohort studies have been undertaken to investigate the associations of total vegetable intake and/or intake of specific vegetables with CVD endpoints [12]. In a recent meta-analysis, Aune et al. [12] showed a summary relative risk (RR) of 0.90 (95%CI 0.87, 0.93) for CVD per 200 g/day increase of vegetable intake. Evidence suggested this relationship was nonlinear (P_nonlinearity_ = 0.04) with steeper inverse associations at lower levels of intake. However, the relationship “appeared” approximately linear with the greatest CVD benefits observed at intakes of 600 g/day, which is slightly higher than most dietary guideline recommendations. For vegetable types, leafy green vegetables, cruciferous vegetables, and tomatoes were inversely associated with CVD risk in nonlinear dose-response analyses. The greatest CVD benefits were observed at intakes of ≥200 g/day for cruciferous vegetable, ≥120 g/day for leafy green vegetables, and ≥200 g/day for tomatoes [12]. However, only a few studies were included in these analyses, therefore results need to be interpreted with caution.

### 5.1. Leafy Green Vegetables

At least 14 studies have reported the associations between intake of leafy green vegetables and CVD [68,69,70,71], atherosclerotic vascular disease (ASVD) [72], coronary heart disease (CHD) [73,74,75], heart disease [76] or stroke [76,77,78,79] (Table 1). The most common vegetables included in the classification of leafy green vegetables were spinach and lettuce. Most studies have demonstrated significant inverse associations between intakes of leafy green vegetables and CVD outcomes [68,71,73,74,76,78]. However, other studies have demonstrated no association [69,70,72,75,76,77,79]. Most positive studies have been conducted in US cohorts [68,71,73,78], with the exception of one in Italy [74] and another in China [76]. Studies demonstrating no associations were conducted in cohorts from Spain [70], Denmark [79], Sweden [77], the Netherlands [75], Japan [69], China [76] and Australia [72].

The US cohorts, Nurses’ Health Study (NHS) and Health Professionals Follow-up Study (HPFS), are the largest cohorts demonstrating an inverse association between intakes of leafy green vegetables and risk of CVD [68], CHD [73] and ischemic stroke [78]. The NHS consists of ~121,700 female registered nurses aged 30–55 years and the NPFS consists of ~51,529 male health professionals aged 40–75 years. Combining these two large cohorts, Hung et al. [68] reported an adjusted RR of 0.89 (95%CI 0.83–0.96) for CVD for every one serving increment of leafy green vegetables. Combining the same cohorts, Bhupathiraju et al. [73] reported an adjusted RR of 0.83 (95%CI 0.77–0.91) for CHD for the highest (median: ~1.5 servings/day) compared with the lowest (median: ~0.2 servings/day) intakes of leafy green vegetables. For ischemic stroke, Joshipura et al. [78] reported an adjusted RR of 0.76 (95%CI 0.58–0.99) for the highest (median: 1.36 servings/day) versus the lowest (median: 0.16 servings/day) intakes. However, the translation of these findings into practice are limited, as the number of grams in one serving was not reported. In a smaller US cohort (*n* = 1273), the Massachusetts Health Care Panel Study (MHCPS), Gaziano et al. [71] reported a RR of 0.49 (95%CI 0.31–0.77) for CVD mortality for those consuming one or more servings per day of salads and leafy green vegetables compared to those consuming less than one serving per day. Again, the number of grams in one serving was not reported, making translation difficult. Importantly, only age and sex were used in the adjusted model, and dietary and lifestyle factors were not taken into consideration. Using the EPICOR study (the European Prospective Investigation into Cancer and Nutrition (EPIC) cohorts in northern (Turin and Varese), central (Florence), and southern (Naples and Ragusa Italy), Bendinelli et al. [74] reported an adjusted hazard ratio (HR) of 0.54 (95%CI 0.33–0.90) for CHD for the highest (>50.8 g/day) compared to the lowest (<17.6 g/day) intakes of leafy green vegetables. This study consisted of 29,689 females aged 35–74 years. Lastly, using the Linxian Nutrition Intervention Trials (NIT) cohort in China, Wang et al. [76] reported an adjusted HR of 0.62 (95%CI 0.43–0.91) for stroke mortality for every twice/week increase in leafy green vegetables. However, this relationship was not observed for heart disease mortality.

### 5.2. Cruciferous Vegetables

At least 26 studies have reported the associations between intake of cruciferous vegetables and CVD [68,69,70,71,80,81,82], ASVD [72], CHD [73,74,75,76,81,83], ischemic heart disease (IHD) [72], heart disease [76], cerebrovascular disease (CVA) [84], ischemic CVA [72], stroke [76,77], ischemic stroke [78,79,84], and intracerebral hemorrhage [84] (Table 2). Broccoli, Brussels sprouts, cabbage, and cauliflower were the most common vegetables grouped as cruciferous vegetables. At least eight studies have identified an inverse relationship between intake of cruciferous vegetables and six separate outcomes: CVD [70,80], ASVD [72], IHD [72], CVA [84], ischemic stroke [78,84] and intracerebral hemorrhage [84]. Seventeen studies reporting five outcomes (CVD, CHD, ischemic CVA, ischemic stroke and stroke) have shown no associations [68,69,71,72,73,74,75,76,77,79,81,82,83].

In the Shanghai Women’s Health Study (SWHS), Zhang et al. [80] reported an adjusted HR of 0.80 (95%CI 0.72–0.89) for CVD for the highest intake (median: 166 g/day) of cruciferous vegetables in comparison to the lowest intake (median: 28 g/day). The SWHS cohort included 74,942 females aged 40–70 years. Similar results were observed in the Shanghai Men’s Health Study (SMHS). An adjusted HR of 0.78 (95%CI 0.71–0.85) was reported for CVD in 61,500 males aged 40–74 years consuming the highest intake (median: 208 g/day) of cruciferous vegetables in comparison to the lowest intake (median: 34 g/day) [80]. No relationship was observed with intake of cruciferous vegetables and CHD in the same cohorts (SWHS and SMHS) [83]. In the PREvención con DIeta MEDiterránea (PREDIMED) study, Buil-Cosiales et al. [70] reported an adjusted HR of 0.64 (95%CI 0.42–0.97) for CVD for the highest cumulative average intake (median: 30 g/day) of cruciferous vegetables in comparison to the lowest cumulative average intake (median: 3.1 g/day). In an Australian cohort, the Perth Longitudinal Study of Ageing Women (PLSAW), we have reported an adjusted HR of 0.88 (95%CI 0.81–0.95) for atherosclerotic vascular disease (ASVD) for every 10 g/day increase in cruciferous vegetables [72]. A similar relationship was evident for IHD (adjusted HR = 0.83, 95%CI 0.75–0.92), but not for ischemic CVA (adjusted HR = 0.94, 95%CI 0.84–1.05) [72]. In the Finnish Mobile Clinic Health Examination Survey (FMCHES) cohort (*n* = 3939), Mizrahi et al. [84] reported an adjusted RR of 0.79 (95%CI 0.63–0.99), 0.67 (95%CI 0.49–0.92), and 0.49 (95%CI 0.25, 0.98) for CVA, ischemic stroke, and intracerebral hemorrhage, respectively. These results were for the highest intakes (men: 14–269 g/day; women: 16–188 g/day) compared to the lowest intakes (men: 0–1 g/day; women: 16–188 g/day) of cruciferous vegetables. In the NHS and NPFS cohorts, highest (median: 1.0 serving/day) versus lowest (median: 0.14 serving/day) intakes of cruciferous vegetables was associated with a lower RR of ischemic stroke (adjusted RR = 0.71, 95%CI 0.55–0.93) [78]. However, a similar relationship was not shown for CVD [68] or CHD [73].

### 5.3. Allium Vegetables

At least 14 studies have investigated the relationships between intake of allium vegetables and ASVD [72], CHD [75,83,85,86], IHD [72,87], CVA [88], ischemic CVA [72], stroke [77], acute stroke [88], ischemic stroke [79], intracerebral hemorrhage [88] and thrombosis or embolia [88] (Table 3). The majority of these studies have investigated the intake of onions. Four studies have reported inverse relationships between intake of allium vegetables and ASVD [72], CHD [86], IHD [72] and ischemic CVA [72] with all other studies reporting no associations [75,77,79,83,85,87,88].

In the PLSAW cohort [72], we have reported an adjusted HR of 0.85 (95%CI 0.75–0.97) for ASVD for every 5 g/day increase in allium vegetable intake. This relationship remained for IHD (adjusted HR = 0.82, 95%CI 0.70–0.97) and ischemic CVA (adjusted HR = 0.75, 95%CI 0.60–0.93). In the Finnish Social Insurance Institution (FSII) cohort, Knekt et al. [86] reported an adjusted RR of 0.50 (95%CI 0.30–0.82) for CHD for women (*n* = 2385) consuming ≥5 g/day compared with <5 g/day of onions. This relationship was not observed in men (*n* = 2748).

### 5.4. Yellow-Orange-Red Vegetables

At least 14 studies have reported associations between yellow-orange-red vegetables and CVD [69,71,81,89,90], ASVD [72], CHD [74,75,85,89], heart disease [76] and stroke [76,77,89] (Table 4). Tomato, carrot, sweet potato and pumpkin were the major vegetables studied. Five studies have reported inverse associations between intake of yellow-orange-red vegetables and CVD [71,89,90], CHD [89] and heart disease [76]. Other studies have reported no associations [69,72,74,75,76,77,81,85,89].

In the Framingham Offspring Study, Jacques et al. [89] reported an inverse relationship for consumption of tomato products (tomatoes, tomato juice and tomato sauce) with an adjusted HR of 0.94 (95%CI 0.88–0.99) for CVD and 0.90 (95%CI 0.83–0.99) for CHD for every one serve increase per day. This relationship was not observed for stroke. In the MHCPS study, Gaziano et al. [71] reported an inverse relationship between consumption of carrots and/or squash and CVD mortality. The RR for those who ate one or more servings per day of carrots and squash was 0.40 (95%CI 0.16–0.98), although the number of grams in one serving was not reported. Furthermore, this relationship was not observed for consumption of tomatoes. In the Zutphen Elderly Study, Buijsse et al. [90] reported an inverse relationship between consumption of carrots and CVD mortality (per SD, RR = 0.83, 95%CI 0.68–1.00). Lastly, in the Linxian NIT study, Wang et al. [76] reported consumption of yellow-orange vegetables, such as sweet potatoes, carrots and pumpkins, was inversely associated with heart disease, but not stroke. The adjusted HR for heart disease was 0.77 (95%CI 0.60–0.97) for every once/day increase in consumption of yellow-orange vegetables.

### 5.5. Legumes

At least 28 studies have reported on the relationships between legume consumption and CVD [68,91,92,93,94], ischemic CVD [95], ASVD [72], CHD [76,83,91,92,93,96,97,98,99,100], CVA [84,101], stroke [76,91,102,103], ischemic stroke [78,84] and intracerebral hemorrhage [84] (Table 5). At least seven studies have reported inverse associations between intakes of legumes and CVD [91,93,94], ischemic CVD [95], CHD [93], heart disease [76], and ischemic stroke [84]. However, some studies have demonstrated no relationship [68,72,76,78,83,84,91,92,96,97,98,99,100,101,102,103].

In the Japanese Collaborative Cohort Study (JCCS), Nagura et al. [91] reported an inverse relationship between bean intake and CVD with an adjusted HR of 0.84 (95%CI 0.74–0.95) for those consuming the highest (median: 4.5 servings/week) compared to the lowest (median: 0.8 servings/week) bean intake. This relationship was not evident for CHD and stroke. In the First National Health and Nutrition Examination Survey Epidemiologic Follow-up Study (NHEFS), Bazzano et al. [93] reported an adjusted RR of 0.89 (95%CI 0.80–0.98) for CVD and 0.78 (95%CI 0.68–0.90) for CHD for those who consumed legumes ≥4 times per week compared to those who consumed legumes less than once per week. In the Isfahan Cohort Study (ICS) in Iran, Nouri et al. [94] reported a 33% lower risk of CVD for old-aged individuals consuming high intakes (>3 times per week) compared to low intakes (0–1 time per week) of legumes (HR = 0.66, 95%CI 0.45–0.98). In the Japan Public Health Centre-Based (JPHC) Study, Kokubo et al. [95] reported an inverse relationship between soy intake and ischemic CVD mortality. The adjusted HR was 0.31 (95%CI 0.13–0.74) for women consuming high intakes (≥5 days per week) compared to low intakes (0–2 days per week) of soy. This relationship was primarily observed in postmenopausal women and was not observed in men or for dietary intakes of beans. Wang et al. [76] reported an inverse relationship between bean intake and heart disease with an adjusted HR of 0.63 (95%CI 0.48–0.83) for increasing bean intake four times/week. This relationship was not observed for stroke. In the FMCHES study, Mizrahi et al. [84] reported an inverse relationship between consumption of legumes and ischemic stroke with an adjusted RR of 0.72 (95%CI 0.54–0.96) for those consuming the highest intakes (men: 10–101 g/day; women: 7–43 g/day) compared to the lowest intakes (men: 0–2 g/day; women: 0–1 g/day). Other related outcomes including CVA and intracerebral hemorrhage were not related.

## 6. Limitations and Future Directions

Although nutrition epidemiology has been successful in providing insight into the potential causes and prevention of many health conditions, several important limitations still exist [104]. Due to the observational nature of nutrition epidemiological studies, residual confounding is inevitable. Challenges also arise in the accuracy of assessing dietary intake [104]. These factors can lead to contradictory findings, especially when comparing results across different populations where dietary assessment methods are sometimes weak and there is large heterogeneity amongst the populations studied. Relationships between vegetable intake and CVD outcomes may be attenuated when large differences in vegetable intake classification and/or categorization exist. There is a need for the international standardization of such variables to limit error, thereby enabling more accurate comparisons between investigations. Such changes if implemented in large cohort studies have the capacity to improve the quality of nutrition research. Technology-based dietary assessment methods in conjunction with recovery biomarkers, such as those that appear in plasma or urine, could be used to improve accuracy and are worth considering in future work [105]. Finally, adopting STROBE-nut guidelines and checklists will improve reporting of nutritional epidemiological studies and the quality of published literature [105,106].

Most prospective studies reported in this review were undertaken in older populations. Greater statistical power is present in studies with more events, which occur more frequently in older populations. Therefore, these populations are frequently selected to study disease-related outcomes such as CVD. It is clear that dietary changes later in life can significantly reduce the risk of chronic disease within five years [107,108]. However, it is likely that the greatest cardiovascular benefit would be to increase vegetable intake throughout the life course. Future studies are needed to evaluate the relationships between intake of specific vegetables and cardiometabolic health outcomes in younger populations. Due to the small number of events that would occur in younger populations, markers of cardiometabolic health are an alternative to study. It is also not known whether the health benefits of increasing vegetable intake is associated with particular racial or ethnic populations, and therefore this should be addressed in future research.

## 7. Conclusions

Many large observational follow-up studies have reported the inverse associations of leafy green, cruciferous, allium, yellow-orange-red vegetables, and legumes with CVD outcomes. These vegetables contain many nutrients and phytochemicals that have been postulated to have cardiovascular health benefits. Some studies demonstrate no associations between specific vegetable types and CVD outcomes. This may be due to type II error and/or bias introduced by measurement error and regression dilution, attenuating observed risk estimates [12]. Other inherit limitations of observational epidemiological studies may also influence relationships. This presents a major limitation of nutritional epidemiology, making the interpretation and comparison of results difficult [109].

The evidence in this review suggests intake of leafy green and cruciferous vegetables may confer strong cardiovascular health benefits. Increasing vegetable intake, with a focus on consuming leafy green and cruciferous vegetables may provide the greatest cardiovascular health benefits. Incorporating such dietary changes along with other recommended lifestyle changes will optimize health benefits. Lifestyle changes include consuming a diet full of vegetable, fruits, and whole grains; including low-fat dairy products, poultry, fish, legumes, non-tropical vegetable oils, and nuts; and limiting intake of saturated and trans fats, sweets, sugar-sweetened beverages, and red meats [110]. Other lifestyle changes include increasing physical activity [110], avoiding cigarette smoking [110] and intake of alcohol [111], and maintaining a healthy body weight by consuming appropriate energy requirements [110]. There is a need for very large well-designed epidemiological studies investigating the cardiovascular health benefits of different vegetable types. Large long-term randomized controlled trials are needed to establish the causal effects of the specific vegetables found to be most beneficial for cardiovascular health.

## Figures and Tables

**Table 1:** Prospective cohort studies of leafy green vegetables and cardiovascular disease outcomes.

Study Cohort (Country)	Sample Number	Sex	Age (years)	Dietary Assessment Method	Outcome	Follow-up (years)	Results	First Author, Year (ref)
NHS and HPFS (USA)	109,635	M and F	30–75	FFQ	CVD (fatal or nonfatal MI or stroke)	12–15	11% ↓ RR (per serving/day)	Hung, 2004 [68]
JPHCPS (Japan)	77,891	M and F	45–74	FFQ	CVD (fatal or nonfatal MI or stroke)	5–8	No association	Takachi, 2007 [69]
PREDIMED (Spain)	7216	M and F	55–80	FFQ	CVD (cardiovascular death, MI or stroke)	7	No association	Buil-Cosiales, 2016 [70]
MHCPS (USA)	1273	M and F	≥66	FFQ	CVD (death)	4.75	51% ↓ RR (≥1 vs. <1 serving/day)	Gaziano, 1995 [71]
PLSAW (Australia)	1226	F	≥70	FFQ	ASVD (fatal ischemic heart disease, heart failure, cerebrovascular disease excluding hemorrhage, or peripheral heart disease)	15	No association	Blekkenhorst, 2017 [72]
NHS (USA)	71,141	F	50 ± 7	FFQ	CHD (fatal CHD or nonfatal MI)	24	22% ↓ RR (high vs. low intake)	Bhupathiraju, 2013 [73]
HPFS (USA)	42,135	M	53 ± 10	FFQ	CHD (fatal CHD or nonfatal MI)	22	12% ↓ RR (high vs. low intake)	Bhupathiraju, 2013 [73]
EPICOR (Italy)	29,689	F	35–74	FFQ	CHD (fatal or nonfatal MI or coronary revascularization)	7.85	46% ↓ HR (high vs. low intake)	Bendinelli, 2011 [74]
MORGEN (The Netherlands)	20,069	M and F	20–65	FFQ	CHD (fatal CHD or nonfatal acute MI)	10	No association	Oude Griep, 2011 [75]
Linxian NIT (China)	2445	M and F	40–69	FFQ	Heart disease (death)	26	No association	Wang, 2016 [76]
SMC and COSM (Sweden)	74,961	M and F	45–83	FFQ	Stroke (cerebral infarction, hemorrhagic stroke or unspecified stroke)	10.2	No association	Larsson, 2013 [77]
Linxian NIT (China)	2445	M and F	40–69	FFQ	Stroke (death)	26	38% ↓ HR (per twice/week)	Wang, 2016 [76]
NHS and HPFS (USA)	114,279	M and F	34–59	FFQ	Ischemic stroke (embolic or thrombotic)	14	84% ↓ RR (high vs. low intake)	Joshipura, 1999 [78]
DDCHS (Denmark)	54,506	M and F	50–64	FFQ	Ischemic stroke (ischemic infarction, intracerebral hemorrhage or subarachnoid hemorrhage)	3.09	No association	Johnsen, 2003 [79]

ASVD, atherosclerotic vascular disease; CHD, coronary heart disease; COSM, Cohort of Swedish Men; CVD, cardiovascular disease; DDCHS, Danish Diet, Cancer, and Healthy Study; EPICOR, European Prospective Investigation into Cancer and Nutrition (EPIC) cohorts in northern (Turin and Varese), central (Florence), and southern (Naples and Ragusa) Italy; F, female; FFQ, food frequency questionnaire; HPFS, Health Professionals Follow-up Study; HR, hazard ratio; JPHCPS, Japan Public Health Centre-based Prospective Study; M, male; MHCPS, Massachusetts Health Care Panel Study; MI, myocardial infarction; MORGEN, Monitoring Project on Risk Factors and Chronic Diseases in The Netherlands; NHS, Nurses’ Health Study; NIT, Nutrition Intervention Trials; PLSAW, Perth Longitudinal Study of Ageing Women; PREDIMED, PREvención con DIeta MEDiterránea study; ref, reference; RR, relative risk; SMC, Swedish Mammography Cohort.

**Table 2:** Prospective cohort studies of cruciferous vegetables and cardiovascular disease outcomes.

Study Cohort (Country)	Sample Number	Sex	Age (years)	Dietary Assessment Method	Outcome	Follow-up (years)	Results	First Author, Year (ref)
NHS and HPFS (USA)	109,635	M and F	30–75	FFQ	CVD (fatal or nonfatal MI or stroke)	12–15	No association	Hung, 2004 [68]
JPHCPS (Japan)	77,891	M and F	45–74	FFQ	CVD (fatal or nonfatal MI or stroke)	5–8	No association	Takachi, 2007 [69]
SWHS (China)	74,942	F	40–70	FFQ	CVD (death)	10.2	20% ↓ HR (high vs. low intake)	Zhang, 2011 [80]
SMHS (China)	61,500	M	40–74	FFQ	CVD (death)	4.6	27% ↓ HR (high vs. low intake)	Zhang, 2011 [80]
IWHS (USA)	34,492	F	55–69	FFQ	CVD (death)	16	No association	Mink, 2007 [81]
PREDIMED (Spain)	7216	M and F	55–80	FFQ	CVD (cardiovascular death, MI or stroke)	7	36% ↓ HR (high vs. low intake)	Buil-Cosiales, 2016 [70]
Odyssey Cohort (USA)	6151	M and F	30–93	FFQ	CVD (death)	14	No association	Genkinger, 2004 [82]
MHCPS (USA)	1273	M and F	≥66	FFQ	CVD (death)	4.75	No association	Gaziano, 1995 [71]
PLSAW (Australia)	1226	F	≥70	FFQ	ASVD (fatal ischemic heart disease, heart failure, cerebrovascular disease excluding hemorrhage, or peripheral heart disease)	15	12% ↓ HR (per 10 g/day)	Blekkenhorst, 2017 [72]
NHS (USA)	71,141	F	50±7	FFQ	CHD (fatal CHD or nonfatal MI)	24	No association	Bhupathiraju, 2013 [73]
SWHS (China)	67,211	F	40–70	FFQ	CHD (fatal CHD or nonfatal MI)	9.8	No association	Yu, 2013 [83]
SMHS (China)	55,474	M	40–75	FFQ	CHD (fatal CHD or nonfatal MI)	5.4	No association	Yu, 2013 [83]
HPFS (USA)	42,135	M	53 ± 10	FFQ	CHD (fatal CHD or nonfatal MI)	22	No association	Bhupathiraju, 2013 [73]
IWHS (USA)	34,492	F	55–69	FFQ	CHD (death)	16	No association	Mink, 2007 [81]
EPICOR (Italy)	29,689	F	35–74	FFQ	CHD (fatal or nonfatal MI or coronary revascularization)	7.85	No association	Bendinelli, 2011 [74]
MORGEN (The Netherlands)	20,069	M and F	20–65	FFQ	CHD (fatal CHD or nonfatal acute MI)	10	No association	Oude Griep, 2011 [75]
PLSAW (Australia)	1226	F	≥70	FFQ	IHD (death)	15	17% ↓ HR (per 10 g/day)	Blekkenhorst, 2017 [72]
Linxian NIT (China)	2445	M and F	40–69	FFQ	Heart disease (death)	26	No association	Wang, 2016 [76]
FMCHES (Finland)	3932	M and F	40–74	FFQ	CVA (fatal or nonfatal)	24	21% ↓ RR (high vs. low intake)	Mizrahi, 2009 [84]
PLSAW (Australia)	1226	F	≥70	FFQ	Ischemic CVA (death)	15	No association	Blekkenhorst, 2017 [72]
SMC and COSM (Sweden)	74,961	M and F	45–83	FFQ	Stroke (cerebral infarction, hemorrhagic stroke or unspecified stroke)	10.2	No association	Larsson, 2013 [77]
Linxian NIT (China)	2445	M and F	40–69	FFQ	Stroke (death)	26	No association	Wang, 2016 [76]
NHS and HPFS (USA)	114,279	M and F	34–59	FFQ	Ischemic stroke (embolic or thrombotic)	14	29% ↓ RR (high vs. low intake)	Joshipura, 1999 [78]
DDCHS (Denmark)	54,506	M and F	50–64	FFQ	Ischemic stroke (ischemic infarction, intracerebral hemorrhage or subarachnoid hemorrhage)	3.09	No association	Johnsen, 2003 [79]
FMCHES (Finland)	3932	M and F	40–74	FFQ	Ischemic stroke (fatal or nonfatal)	24	33% ↓ RR (high vs. low intake)	Mizrahi, 2009 [84]
FMCHES (Finland)	3932	M and F	40–74	FFQ	Intracerebral hemorrhage (fatal or nonfatal)	24	51% ↓ RR (high vs. low intake)	Mizrahi, 2009 [84]

ASVD, atherosclerotic vascular disease; CHD, coronary heart disease; COSM, Cohort of Swedish Men; CVA, cerebrovascular disease; CVD, cardiovascular disease; DDCHS, Danish Diet, Cancer, and Healthy Study; EPICOR, European Prospective Investigation into Cancer and Nutrition (EPIC) cohorts in northern (Turin and Varese), central (Florence), and southern (Naples and Ragusa) Italy; F, female; FFQ, food frequency questionnaire; FMCHES, Finnish Mobile Clinic Health Examination Survey; HPFS, Health Professionals Follow-up Study; HR, hazard ratio; IHD, ischemic heart disease; IWHS, Iowa Women’s Health Study; JPHCPS, Japan Public Health Centre-based Prospective Study; M, male; MHCPS, Massachusetts Health Care Panel Study; MI, myocardial infarction; MORGEN, Monitoring Project on Risk Factors and Chronic Diseases in The Netherlands; NHS, Nurses’ Health Study; NIT, Nutrition Intervention Trials; PLSAW, Perth Longitudinal Study of Ageing Women; PREDIMED, PREvención con DIeta MEDiterránea study; ref, reference; RR, relative risk; SMC, Swedish Mammography Cohort; SMHS, Shanghai Men’s Health Study; SWHS, Shanghai Women’s Health Study.

**Table 3:** Prospective cohort studies of allium vegetables and cardiovascular disease outcomes.

Study Cohort (Country)	Sample Number	Sex	Age (years)	Dietary Assessment Method	Outcome	Follow-up (years)	Results	First Author, Year (ref)
PLSAW (Australia)	1226	F	≥70	FFQ	ASVD (fatal ischemic heart disease, heart failure, cerebrovascular disease excluding hemorrhage, or peripheral heart disease)	15	15% ↓ HR (per 5 g/day)	Blekkenhorst, 2017 [72]
SWHS (China)	67,211	F	40–70	FFQ	CHD (fatal CHD or nonfatal MI)	9.8	No association	Yu, 2013 [83]
NHS (USA)	66,360	F	30–55	FFQ	CHD (fatal CHD or nonfatal MI)	12	No association	Lin, 2007 [85]
SMHS (China)	55,474	M	40–75	FFQ	CHD (fatal CHD or nonfatal MI)	5.4	No association	Yu, 2013 [83]
MORGEN (The Netherlands)	20,069	M and F	20–65	FFQ	CHD (fatal CHD or nonfatal acute MI)	10	No association	Oude Griep, 2011 [75]
FSII (Finland)	2748	M	30–69	DHQ	CHD (death)	26	No association	Knekt, 1996 [86]
FSII (Finland)	2385	F	30–69	DHQ	CHD (death)	26	50% ↓ RR (high vs. low intake)	Knekt, 1996 [86]
Caerphilly Study (UK)	2512	M	45–59	FFQ	IHD (IHD death, nonfatal MI, MI define by electrocardiogram)	10	No association	Hertog, 1997 [87]
PLSAW (Australia)	1226	F	≥70	FFQ	IHD (death)	15	18% ↓ HR (per 5 g/day)	Blekkenhorst, 2017 [72]
FMCHES (Finland)	9208	M and F	≥15	DHQ	CVA (fatal or nonfatal)	28	No association	Knekt, 2000 [88]
PLSAW (Australia)	1226	F	≥70	FFQ	Ischemic CVA (death)	15	25% ↓ HR (per 5 g/day)	Blekkenhorst, 2017 [72]
SMC and COSM (Sweden)	74,961	M and F	45–83	FFQ	Stroke (cerebral infarction, hemorrhagic stroke or unspecified stroke)	10.2	No association	Larsson, 2013 [77]
FMCHES (Finland)	9208	M and F	≥15	DHQ	Acute strokes	28	No association	Knekt, 2000 [88]
DDCHS (Denmark)	54,506	M and F	50–64	FFQ	Ischemic stroke (ischemic infarction, intracerebral hemorrhage or subarachnoid hemorrhage)	3.09	No association	Johnsen, 2003 [79]
FMCHES (Finland)	9208	M and F	≥15	DHQ	Intracerebral hemorrhage	28	No association	Knekt, 2000 [88]
FMCHES (Finland)	9208	M and F	≥15	DHQ	Thrombosis or embolia	28	No association	Knekt, 2000 [88]

ASVD, atherosclerotic vascular disease; CHD, coronary heart disease; COSM, Cohort of Swedish Men; CVA, cerebrovascular disease; DDCHS, Danish Diet, Cancer, and Healthy Study; DHQ, dietary history questionnaire; F, female; FFQ, food frequency questionnaire; FMCHES, Finnish Mobile Clinic Health Examination Survey; FSII, Finnish Social Insurance Institution; HR, hazard ratio; IHD, ischemic heart disease; M, male; MI, myocardial infarction; MORGEN, Monitoring Project on Risk Factors and Chronic Diseases in The Netherlands; NHS, Nurses’ Health Study; PLSAW, Perth Longitudinal Study of Ageing Women; ref, reference; RR, relative risk; SMC, Swedish Mammography Cohort; SMHS, Shanghai Men’s Health Study; SWHS, Shanghai Women’s Health Study.

**Table 4:** Prospective cohort studies of yellow-orange-red vegetables and cardiovascular disease outcomes.

Study Cohort (Country)	Sample Number	Sex	Age (years)	Dietary Assessment Method	Outcome	Follow-up (years)	Results	First Author, Year (ref)
JPHCPS (Japan)	77,891	M and F	45–74	FFQ	CVD (MI or stroke)	5–8	No association	Takachi, 2007 [69]
IWHS (USA)	34,489	F	55–69	FFQ	CVD (CHD or stroke)	16	No association	Mink, 2007 [81]
Framingham Offspring Study (USA)	2525	M and F	26–79	FFQ	CVD (fatal or nonfatal CHD, CVA, congestive heart failure or peripheral vascular disease)	11	6% ↓ HR (per 1 serving/day)	Jacques, 2013 [89]
MHCPS (USA)	1273	M and F	≥66	FFQ	CVD (death)	4.75	60% ↓ RR (≥1 vs. <1 serving)	Gaziano, 1995 [71]
Zutphen Elderly Study (The Netherlands)	559	M	65–84	DHQ	CVD (fatal ischemic heart disease, stroke or other diseases of the circulatory system)	15	17% ↓ RR (per SD)	Buijsse, 2008 [90]
PLSAW (Australia)	1226	F	≥70	FFQ	ASVD (fatal ischemic heart disease, heart failure, cerebrovascular disease excluding hemorrhage, or peripheral heart disease)	15	No association	Blekkenhorst, 2017 [72]
NHS (USA)	66,360	F	30–55	FFQ	CHD (fatal CHD or nonfatal MI)	12	No association	Lin, 2007 [85]
EPICOR (Italy)	29,689	F	35–74	FFQ	CHD (fatal or nonfatal MI or coronary revascularization)	7.85	No association	Bendinelli, 2011 [74]
MORGEN (The Netherlands)	20,069	M and F	20–65	FFQ	CHD (nonfatal acute MI or fatal CHD)	10	No association	Oude Griep, 2011 [75]
Framingham Offspring Study (USA)	2525	M and F	26–79	FFQ	CHD (MI, angina pectoris, coronary insufficiency or CHD death)	11	10% ↓ HR (per 1 serving/day)	Jacques, 2013 [89]
Linxian NIT (China)	2445	M and F	40–69	FFQ	Heart disease (death)	26	23% ↓ HR (per once/day)	Wang, 2016 [76]
SMC and COSM (Sweden)	74,961	M and F	45–83	FFQ	Stroke (cerebral infarction, hemorrhagic stroke or unspecified stroke)	10.2	No association	Larsson, 2013 [77]
Framingham Offspring Study (USA)	2525	M and F	26–79	FFQ	Stroke (nonfatal)	11	No association	Jacques, 2013 [89]
Linxian NIT (China)	2445	M and F	40–69	FFQ	Stroke (death)	26	No association	Wang, 2016 [76]

ASVD, atherosclerotic vascular disease; CHD, coronary heart disease; CVD, cardiovascular disease; DHS, dietary history questionnaire; EPICOR, European Prospective Investigation into Cancer and Nutrition (EPIC) cohorts in northern (Turin and Varese), central (Florence), and southern (Naples and Ragusa) Italy; F, female; FFQ, food frequency questionnaire; HR, hazard ratio; IWHS, Iowa Women’s Health Study; JPHCPS, Japan Public Health Centre-based Prospective Study; M, male; MHCPS, Massachusetts Health Care Panel Study; MI, myocardial infarction; MORGEN, Monitoring Project on Risk Factors and Chronic Diseases in The Netherlands; NHS, Nurses’ Health Study; NIT, Nutrition Intervention Trials; PLSAW, Perth Longitudinal Study of Ageing Women; ref, reference; RR, relative risk.

**Table 5:** Prospective cohort studies of legumes and cardiovascular disease outcomes.

Study Cohort (Country)	Sample Number	Sex	Age (years)	Dietary Assessment Method	Outcome	Follow-up (years)	Results	First Author, Year (ref)
NHS and HPFS (USA)	109,635	M and F	30–75	FFQ	CVD (fatal or nonfatal MI or stroke)	12–15	No association	Hung, 2004 [68]
JCCS (Japan)	59,485	M and F	40–79	FFQ	CVD (death)	12.7	16% ↓ HR (high vs. low intake)	Nagura, 2009 [91]
The SUN Project (Spain)	13,609	M and F	38	FFQ	CVD (cardiovascular death, MI, revascularization procedures, fatal or nonfatal stroke)	4.9	No association	Martínez-González, 2011 [92]
NHEFS (USA)	9632	M and F	25–74	FFQ	CVD (fatal or nonfatal)	21	11% ↓ RR (≥4 vs. <1 times/week)	Bazzano, 2001 [93]
ICS (Iran)	6504	M and F	≥35	FFQ	CVD (fatal or nonfatal MI, sudden cardiac death, unstable angina or stroke)	6.8	33% ↓ HR (>55 years only) (high vs. low intake)	Nouri, 2016 [94]
JPHC (Japan)	40,462	M and F	40–59	FFQ	Ischemic CVD (fatal CI or MI)	13	69% ↓ HR (F only) (high vs. low intake)	Kokubo, 2007 [95]
PLSAW (Australia)	1226	F	≥70	FFQ	ASVD (fatal ischemic heart disease, heart failure, cerebrovascular disease excluding hemorrhage, or peripheral heart disease	15	No association	Blekkenhorst, 2017 [72]
IWHS (USA)	99,826	F	55–69	FFQ	CHD (death)	15	No association	Kelemen, 2005 [96]
NHS (USA)	84,136	F	30–55	FFQ	CHD (fatal CHD or nonfatal MI)	26	No association	Bernstein, 2010 [97]
SWHS (China)	67,211	F	40–70	FFQ	CHD (fatal CHD or nonfatal MI)	9.8	No association	Yu, 2013 [83]
JCCS (Japan)	59,485	M and F	40–79	FFQ	CHD (death)	12.7	No association	Nagura, 2009 [91]
SMHS (China)	55,474	M	40–75	FFQ	CHD (fatal CHD or nonfatal MI)	5.4	No association	Yu, 2013 [83]
EPIC (Spain)	41,078	M and F	29–69	FFQ	CHD (fatal or nonfatal)	10.4	No association	Buckland, 2009 [98]
EPIC (Greece)	23,929	M and F	20–86	FFQ	CHD (fatal or nonfatal MI, angina or other CHD)	10	No association	Dilis, 2012 [99]
The SUN Project (Spain)	13,609	M and F	38	FFQ	CHD (fatal)	4.9	No association	Martínez-González, 2011 [92]
ARIC (USA)	12,066	M and F	45–64	FFQ	CHD (fatal CHD or nonfatal MI)	22	No association	Haring, 2014 [100]
NHEFS (USA)	9632	M and F	25–74	FFQ	CHD (fatal or nonfatal)	21	22% ↓ RR (≥4 vs. <1 time/week)	Bazzano, 2001 [93]
Linxian NIT (China)	2445	M and F	40–69	FFQ	Heart disease (death)	26	37% ↓ HR (4 times/week)	Wang, 2016 [76]
EPIC (Greece)	23,601	M and F	25–67	FFQ	CVA (fatal or nonfatal)	10.6	No association	Misirli, 2012 [101]
FMCHES (Finland)	3932	M and F	40–74	FFQ	CVA (fatal or nonfatal)	24	No association	Mizrahi, 2009 [84]
NHS (USA)	84,010	F	30–55	FFQ	Stroke (fatal or nonfatal ischemic, hemorrhagic or other stroke)	26	No association	Bernstein, 2012 [102]
JCCS (Japan)	59,485	M and F	40–79	FFQ	Stroke (fatal ischemic or hemorrhagic stroke)	12.7	No association	Nagura, 2009 [91]
HPFS (USA)	43,150	M	40–75	FFQ	Stroke (fatal or nonfatal ischemic, hemorrhagic or other stroke)	22	No association	Bernstein, 2012 [102]
ARIC (USA)	11,601	M and F	45–64	FFQ	Stroke (fatal or nonfatal ischemic or hemorrhagic stroke)	22.7	No association	Haring, 2015 [103]
Linxian NIT (China)	2445	M and F	40–69	FFQ	Stroke (death)	26	No association	Wang, 2016 [76]
NHS and HPFS (USA)	114,279	M and F	34–59	FFQ	Ischemic stroke (embolic or thrombotic)	14	No association	Joshipura, 1999 [78]
FMCHES (Finland)	3932	M and F	40–74	FFQ	Ischemic stroke (fatal or nonfatal)	24	28% ↓ RR (high vs. low intake)	Mizrahi, 2009 [84]
FMCHES (Finland)	3932	M and F	40–74	FFQ	Intracerebral hemorrhage (fatal or nonfatal)	24	No association	Mizrahi, 2009 [84]

ARIC, Atherosclerosis Risk in Communities; ASVD, atherosclerotic vascular disease; CHD, coronary heart disease; CI, cerebral infarction; CVA, cerebrovascular disease; CVD, cardiovascular disease; EPIC, European Prospective Investigation into Cancer and Nutrition; F, female; FFQ, food frequency questionnaire; FMCHES, Finnish Mobile Clinic Health Examination Survey; HPFS, Health Professionals Follow-up Study; HR, hazard ratio; ICS, Isfahan Cohort Study; IWHS, Iowa Women’s Health Study; JCCS, Japanese Collaborative Cohort Study; JPHC, Japan Public Health Centre-Based Study; M, male; MI, myocardial infarction; NHEFS, First National Health and Nutrition Examination Survey Epidemiologic Follow-up Study; NHS, Nurses’ Health Study; NIT, Nutrition Intervention Trials; PLSAW, Perth Longitudinal Study of Ageing Women; ref, reference; SMHS, Shanghai Men’s Health Study; SUN, Seguimiento Universidad de Navarra; SWHS, Shanghai Women’s Health Study.
